# Analysis of Community Composition of Bacterioplankton in Changle Seawater in China by Illumina Sequencing Combined with Bacteria Culture

**DOI:** 10.1111/os.13060

**Published:** 2021-11-24

**Authors:** Du Wang, Qingcong Zheng, Qi Lv, Yuanqing Cai, Yun Zheng, Huidong Chen, Wenming Zhang

**Affiliations:** ^1^ Department of Joint Surgery the First Affiliated Hospital of Fujian Medical University Fuzhou China; ^2^ Department of Orthopedics 900th Hospital of Joint Logistics Support Force Fuzhou China

**Keywords:** Bacteria culture, Bacterioplankton, Community composition, High‐throughput sequencing, Illumina MiSeq

## Abstract

**Objectives:**

To characterize the abundance and relative composition of seawater bacterioplankton communities in Changle city using Illumina MiSeq sequencing and bacterial culture techniques.

**Methods:**

Seawater samples and physicochemical factors were collected from the coastal zone of Changle city on 8 September 2019. Nineteen filter membranes were obtained after using a suction filtration system. We randomly selected eight samples for total seawater bacteria (SWDNA group) sequencing and three samples for active seawater bacteria (SWRNA group) sequencing by Illumina MiSeq. The remaining eight samples were used for bacterial culture and identification. Alpha diversity including species coverage (Coverage), species diversity (Shannon–Wiener and Simpson index), richness estimators (Chao1), and abundance‐based richness estimation (ACE) were calculated to assess biodiversity of seawater bacterioplankton. Beta diversity was used to evaluate the differences between samples. The species abundance differences were determined using the Wilcoxon rank‐sum test. Statistical analyses were performed in R environment.

**Results:**

The Alpha diversity in the SWDNA group in each index was ACE 3206.99, Chao1 2615.12, Shannon 4.64, Simpson 0.05, and coverage 0.97; the corresponding index was ACE 1199.55, Chao1 934.75, Shannon 3.49, Simpson 0.09, and coverage 0.99. The sequencing results of seawater bacterial genes in the coastal waters of Changle city showed that the phyla of high‐abundance bacteria of both the SWDNA and SWRNA groups included *Cyanobacteria*, *Proteobacteria*, and *Bacteroidetes*. The main classes included *Oxyphotobacteria*, *Alphaproteobacteria*, and *Gammaproteobacteria*. The main genera included *Synechococcus CC9902*, *Chloroplast*, and *Cyanobium_PCC‐6307*. Beta diversity analysis showed a significant difference between the SWDNA and SWRNA groups (*P* < 0.05). The species abundance differences between SWDNA and SWRNA groups after Wilcoxon rank‐sum test showed that, at the phylum level, *Actinomycetes* was more abundant in SWDNA group (9.17 *vs* 1.02%, *P* < 0.05); at the class level, *Actinomycetes* (*δ‐ Proteus*) was more abundant in SWDNA group (9.47% *vs* 1.01%, *P* < 0.05); and at the genus level, *Chloroplast* was more abundant in SWRNA group (13.07% *vs* 44.57%, *P* < 0.05). Nine species and 53 colonies were found by bacterial culture: 20 strains of *Vibrio* (37.74%), 22 strains of *Enterobacter* (41.51%), and 11 strains of non‐fermentative bacteria (20.75%).

**Conclusion:**

Illumi MiSeq sequencing of seawater bacteria revealed that the total bacterial community groups and the active bacterial community groups mainly comprised *Cyanobacteria*, *Proteobacteria*, and *Bacteroides* at the phylum level; *Oxyphotobacteria*, *α‐Proteobacteria*, and *γ‐Proteobacteria* at the class level; with *Synechococcus_CC9902*, *Chloroplast*, and *Cyanobium_PCC‐6307* comprising the predominant genera. Exploring the composition and differences of seawater bacteria assists understanding regarding the biodiversity and the infections related to seawater bacteria along the coast of the Changle, provides information that will aid in the diagnosis and treatment of such infections.

## Graphical abstract

In the SWDNA and SWRNA groups, the dominance of the phyla were *Cyanobacteria*, *Proteobacteria*, and *Bacteroides*; the main classes were *oxygen‐producing photobacteria*, *α‐proteobacteria*, and *γ‐proteobacteria*. The predominant genera are *Synechococcus_CC9902*, *Chloroplast*, *Cyanobium_PCC‐6307*, *Candidatus Actinomarina*, *Ascidiaceihabitans* and *HIMB11* in SWDNA; *Chloroplast*, *Synechococcus_CC9902*, *HIMB11*, *Cyanobium_PCC‐6307*, *Ascidiaceihabitans*, and *Ralstonia* in SWRNA.

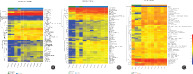

## Introduction

The ocean is the origin of life on earth and contains rich and colorful microbes. Microbial communities are complex, highly diverse, and extremely important, and it is speculated that the ocean is thought to contain approximately 1 × 10^29^ bacteria that have been evolving for over than 3.5 billion years^1^. Bacteria and Archaea numerically dominate the microbial fraction, including both autotrophic primary producers that carry out photosynthesis and heterotrophic organisms that recycle the dissolved organic carbon and nutrients. Bacteria in the ocean are halophilic, which is the most common feature of marine microorganisms: a high‐sodium and hypertonic environment is necessary for their growth. Several studies show that differences of biotic and abiotic factors such as salinity, temperature, oxygen, depth, trophic status, and geographic location^2^, ^3^, ^4^, ^5^, ^6^ contribute to the diversity of marine bacteria. The Global Ocean Survey, which obtained 7.7 mn sequences, showed that 57% of sequences were unique at an identity cutoff of 98%, suggesting a high degree of heterogeneity in the oceans. In the Mediterranean Sea coastal zone, the dominant bacteria are *Proteobacteria* (69%) and *Bacterodetes* (27%); *Cyanobacteria* only accounts for 1% of bacteria^7^. A study conducted in the western English Channel reported that the most abundant phylum was *Proteobacteria*, accounting for 50.3%^8^. Zhao *et al*.^9^ obtained 652,037 high‐quality sequences from 19 sites in Bohai Bay in China and reported that the predominant bacterial phyla were *Gamma‐proteobacteria* (40.49%), *Alpha‐proteobacteria* (22.53%), and *Bacteroidetes* (19.94%). Another study conducted in the South Sea of Korea shows that the phylum *Proteobacteria* was the predominant phylum in all samples, accounting for 67.9% of total bacterial sequences, followed by *Bacteroidetes*, *Cyanobacteria*, *Actinobacteria*, and *Marinimicrobia* (13.9, 4.9, 4.5, and 1.6%, respectively)^10^. In contrast, Campbell *et al*.^11^ reported that the most abundant bacteria in southeast Florida were *Cyanobacteria*, *Bacteroidetes*, *and Proteobacteria*.

While the majority of these ocean bacteria are harmless, several species can potentially infect humans. These bacteria enter the body through seafood or wounds, causing disease and death every year^12^, ^13^, ^14^, ^15^, ^16^. In the United States, annually, it is reported that an estimated 8000 people experience an illness caused by *Vibrio*, a common genus of microorganism in the ocean^17^. In a survey conducted by Hlady and Klontz^18^, 6.5% of wound infections were found to be caused by *Vibrio cholerae* non‐O1 strains, resulting in a case fatality rate of 5%. Hence, an improved understanding concerning the composition of ocean bacterioplankton communities may help to improve diagnosis and treatment of these diseases. It is therefore necessary to investigate composition of bacterioplankton communities.

Changle city is located in Fujian province in the southeast of China, and the coastal zone of Changle city belongs to the East China Sea (ECS), which is located in the northwestern region of the Pacific Ocean and has an area of 7.7 × 10^5^ km^2^ and a depth of 77 m; there are four rivers flow into the ECS: the Changjiang River, Qiantang River, Oujiang River, and Minjiang River^19^. The surface currents of ECS are composed of the northward Taiwan Warm Current and the relatively colder Fujian‐Zhejiang Coastal Current southeast of China^20^. The organic matter input from the Yangtze River and other rivers is washed into the Okinawa Trench under the action of the near‐bottom stream, which is then engulfed by the Taiwan Warm Current and becomes part of the organic matter cycle of the ECS^21^. Overall, the ECS has complex ocean currents, strong ocean‐land interactions, and active biological activities, making the ECS an ideal area for studying ocean microbes.

Compared with the traditional method of plate culture, culture‐independent molecular analysis has become commonplace, since most microbial diversity is unculturable. With the aid of emerging technologies, such as high‐throughput sequencing, it contributing to a more detailed understanding of microbial assemblages which were also called microbiome, including their potential functions^22^. Early microbiology projects, such as the Sargasso Sea Sequencing Project^23^ and its larger branch the Global Ocean sampling Expedition^24^, expanded the understanding of the marine microorganisms through the discovery of new system types and functional genes^25^. With the improvement of sequencing capabilities, such as Illumina MiSeq sequencing, other studies, such as the Human Microbiology Project conducted by Human Microbiome Consortium in 2012^26^, and the Earth Microbiology Project^27^ have described microbial diversity patterns in different hosts and habitats, revealing the non‐random distribution of microbial groups in space or time^28^. However, at present, little is known about the composition of bacterioplankton communities of ECS in China, especially in the coastal zone of Changle city.

The aim of our study is to: (i) assess the physicochemical characteristics of the seawater environment in the coastal zone of Changle city; (ii) assess the composition of the total bacterial community and active bacterial community in phylum, class, and genus levels using Illumina MiSeq sequencing; and (iii) combine bacterial culture technology to identify species of ocean bacteria to better characterize the abundance and composition of bacterioplankton communities in the coastal zone of Changle city.

## Materials and Methods

### Site and Sampling Description

Forty samples of 500 mL seawater were collected from the coastal zone of Xiangbi Bay, which is located in Changle City, Fujian province (25.9633°N, 119.7104°E). All sampling sites were collected at a depth of 0.5 m and 50 m from the coast on 8 September 2019. Samples were immediately stored in a black light‐proof bucket and sent to the laboratory for analysis within 2 h. Meanwhile, physicochemical factors such as the seawater temperature; pH; osmotic pressure; Na^+^, K^+^, and Ca^2+^ concentration were measured using a multi‐parameter water quality detector (JY‐500D, China).

Bacterioplankton enrichment was conducted by a suction filtration system with filter membranes (pore diameter 0.22 μm, diameter 50 mm, mixed cellulose esters, China), and 19 seawater membranes were finally obtained from 40 samples. We randomly selected eight samples for total seawater bacteria sequencing and three samples for active seawater bacteria sequencing; moreover, eight remaining samples were used for bacterial culture and identification.

### DNA and RNA Extraction and PCR Amplification

The extraction of DNA from Bacterioplankton collected from the filter membrane was conducted using the E.Z.N.A.® Soil DNA kit (Omega Bio‐tek, Norcross, GA, USA) following the manufacturer's protocols. Further, the quality of DNA samples was assessed using 1.0% agarose gel electrophoresis, and the DNA concentration was measured using a NanoDrop spectrophotometer.

Hypervariable regions V3–V4 of bacterial 16S rRNA genes were amplified using barcoded adaptor and primer 338F (5′‐ACTCCTACGGGAGGCAGCAG′) and 806R (5′‐GGACTACHVGGGTWTCTAAT‐3′)^29^, ^30^. Polymerase chain reactions (PCR) were performed in triplicate in 20‐μL reactions, containing 4 μL 5*FastPfu buffer, 2 μL 2.5 mM dNTPs, 0.8 μL primer (5 μM), 0.4 μL FastPfu polymerase, and 10 ng DNA template. Reaction conditions consisted of an initial denaturation at 95°C for 3 min, followed by 27 cycles of 95°C for 30 s, 55°C for 30 s, and 72°C for 30 s, and then a final extension was carried out at 72°C for 10 min.

After removing the DNA, complementary DNA (cDNA) (Tiangen, Beijing, China) was synthesized from the extracted RNA as the bacterial 16S rRNA transcript. Similarly, the primers 338F/806R targeted the V3–V4 region to amplify cDNA following the same steps described above^31^, ^32^, ^33^, ^34^.

### Illumina MiSeq Sequencing

A 2% agarose gel was used to extract amplicons, which were purified using an AxyPrep DNA Gel Extraction Kit (AxygenBiosciences, Union City, CA, USA), and quantified with QuantiFluor™‐ST (Promega‐GloMax Promega, Madison, Wisconsin, USA). According to the standard operating procedures of the MiSeq platform (Illumina, San Diego, CA, USA), the purified amplified fragments were pooled in equimolar concentrations and sequenced using 300 bp paired‐end model^35^, ^36^.

Sequencing paired‐end reads from the original DNA/cDNA fragments were trimmed and merged using Fast Length Adjustment of Short reads (FLASH) software (version 1.2.11) as follows: (i) set in 50‐bp windows and if the average quality value in the window was less than 20, the base from the front of the window was cut off and bases less than 50 bp were removed after quality control; (ii) the maximum mismatch rate between overlaps was 0.2 in the splicing process, and the minimum length was 10 bp; and (iii) according to the barcode and primers at the beginning and end of the sequence, the sequence was divided into each sample. The barcode needed to be matched accurately. Sequences with ambiguous bases were removed.

The UPARSE algorithm (version 7.1) was used to perform operational taxonomic units (OTU) clustering of sequences based on 97% similarity cut‐off and UCHIME was used to remove single sequences and chimeras during the clustering process^37^. RDP Classifier was used to analyze and compare against the Silva database (SSU132) using a threshold of 70%^38^, ^39^.

### Bacterial Culture, Isolation, and Identification

Eight seawater filter membranes were randomly placed in different enrichment solutions. After aseptic procedure, two filter membranes were added into two 25 mL containers of alkaline peptone water by inoculation loop for enrichment, and cultured at 35°C for 6–8 h. Afterwards, the surface culture was inoculated with thiosulfate citrate bile salts sucrose agar (TCBS) agar and 3% NaCl tryptic soy agar at 35°C for 18–24 h, respectively.

Two filter membranes were added into two 25 mL Rappaport‐Vassiliadis Soya Broth by inoculation loop for enrichment, and cultured at 35°C for 18–24 h. Afterwards, the surface culture was inoculated with Salmonella‐Shigella (SS) agar and China blue agar at 35°C for 18–24 h.

Three filter membranes were added into 25 mL nutrient broth by inoculation loop for enrichment, and cultured at 35°C for 12–18 h. Afterwards, the surface culture was inoculated into blood agar, chocolate agar, and MacConkey agar at 35°C for 18–24 h.

One filter membrane was added into 19 mL Giolitti and Cantoni Broth by inoculation loop for enrichment, sealed with paraffin and cultured at 35°C for 48 h. After that the surface culture was inoculated into Baird‐Parker agar for 24–48 h.

In the above eight media, the bacterias on blood agar plate, McConkey agar plate, chocolate plate, and China blue plate were identified according to *Vibrio*, *Enterobacter*, *non‐fermentative bacteria*, *Staphylococci* and Gram‐positive *bacilli*; 3% NaCl tryptic soy agar, TCBS, SS plate, and Baird‐Parker plate were identified according to *Vibrio*, *Vibrio*, *Enterobacter* and *Staphylococcus*, respectively. All bacteria on the plates were counted after identification.

### Evaluation criteria

#### Alpha Diversity

Alpha diversity refers to the diversity within each sample. Alpha diversity estimates by multiple indices, including species richness, which is an index that represents the number of different species in a sample and is calculated by Chao1 and abundance‐based richness estimation (ACE); species diversity, which refers to how evenly the microbes distribute in a sample, is calculated by Shannon–Wiener and Simpson index; and the coverage index, which gives the number of groups needed to have a given proportion of the samples occupied. Alpha diversity was calculated using mothur (v1.30.2, https://mothur.org/), an open‐source and community‐supported software.

#### Beta Diversity

Beta diversity shows the difference between microbial communities from different samples and mainly focuses on the difference in taxonomic abundance profiles from different samples. In our study, beta diversity was calculated by principal coordinates analysis (PCoA), which is performed to evaluate the overall differences in microbial community structure based on weighted UniFrac distances and Bray–Curtis distances. An analysis of similarities (ANOSIM) was then employed to test the significance of differences among the bacterioplankton community composition.

### Statistical Analysis

Statistical analyses were performed in R environment (v3.2.2, Lucent Technologies Inc., Murray Hill, NJ, USA), using vegan and ade4 packages with normalized sequences. The species abundance differences were determined using the Wilcoxon rank‐sum test. Statistical significance was inferred by *P* < 0.05.

## Results

### Physicochemical Characteristics of the Seawater Environment

In our study, average ambient temperature was 28.6 ± 1.3 °C and average seawater temperature was 29.1 ± 3.2 °C along the coast. Average temperature was 25.0 ± 0.5 °C and average water temperature was 28.5 ± 1.4 °C in laboratory circumstance. Sea water ion concentration including sodium ion was 325 mmol/L, chloride was 378 mmol/L, potassium ion was 8.3 mmol/L, and calcium ion was 9.1 mmol/L. PH and osmotic pressure values of seawater were 8.0316 ± 0.4 and 725, respectively (Table S1).

### Analysis of the Diversity and Richness of OTU

Eight samples were used for total bacterial high‐throughput sequencing (16S rDNA sequencing, which including live and dead bacteria); three samples were used for seawater active bacteria high‐throughput sequencing (16S rRNA sequencing, only live bacteria were detected). For the SWDNA group, there were 439,410 high‐quality bacterial V3–V4 sequences; the optimized reads for each sample ranged from 50,123 to 63,828. For the SWRNA group, there were 133,137 high‐quality sequences; the optimized reads for each sample ranged from 27,147 to 53,133.

The average number of samples in the SWDNA group was OTU 429.50, ACE 3206.99, Chao 2615.12, Shannon 4.64, Simpson 0.05, and coverage 0.97; the average number of samples in the SWRNA group was OTU 350.67, ACE 1199.55, Chao 934.75, Shannon 3.49, Simpson 0.09, and coverage 0.99. It revealed that the Shannon index of the SWDNA group was higher than that of the SWRNA group, while the Simpson index was opposite and the Ace and Chao indexes of the SWDNA group were higher than those of the SWRNA group. These data show that the bacterial diversity and richness of the SWDNA group samples were higher than the SWRNA group (Table 1).

**TABLE 1:** OTUS number, community richness index (Chao and Ace), coverage rate and community diversity index (Shannon and Simpson) of each group of samples

Sample ID	Sequences	97%					
		Sobs	Shannon	Simpson	Ace	Chao	Coverage
SWDNA1	50,123	1840	4.783345	0.040793	3426.103801	2779.330357	0.970715
SWDNA2	63,828	1977	5.056559	0.031514	3021.590045	2911.400552	0.969684
SWDNA3	48,572	1899	5.020008	0.034073	3598.112957	2912.149533	0.970273
SWDNA4	52,950	1421	4.009747	0.077293	2880.162936	2201.719557	0.976019
SWDNA5	57,901	1722	4.995356	0.024816	3444.001983	2789.751799	0.971599
SWDNA6	53,765	1494	4.179343	0.069658	3193.475475	2542.876033	0.973736
SWDNA7	52,726	1428	4.247337	0.055843	3059.630591	2398.506438	0.975209
SWDNA8	59,545	1517	4.815325	0.029095	3032.859009	2385.225191	0.975135
SWRNA9	53,133	592	3.158448	0.107221	1477.686948	1096.879518	0.989317
SWRNA10	52,857	516	3.040817	0.120905	1461.157539	1038	0.990386
SWRNA11	27,147	621	4.274405	0.054386	659.793709	669.358209	0.997016

### Composition Analysis of the Total Bacterial Community and Active Bacterial Community

#### Phylum Level

**Fig 1:**
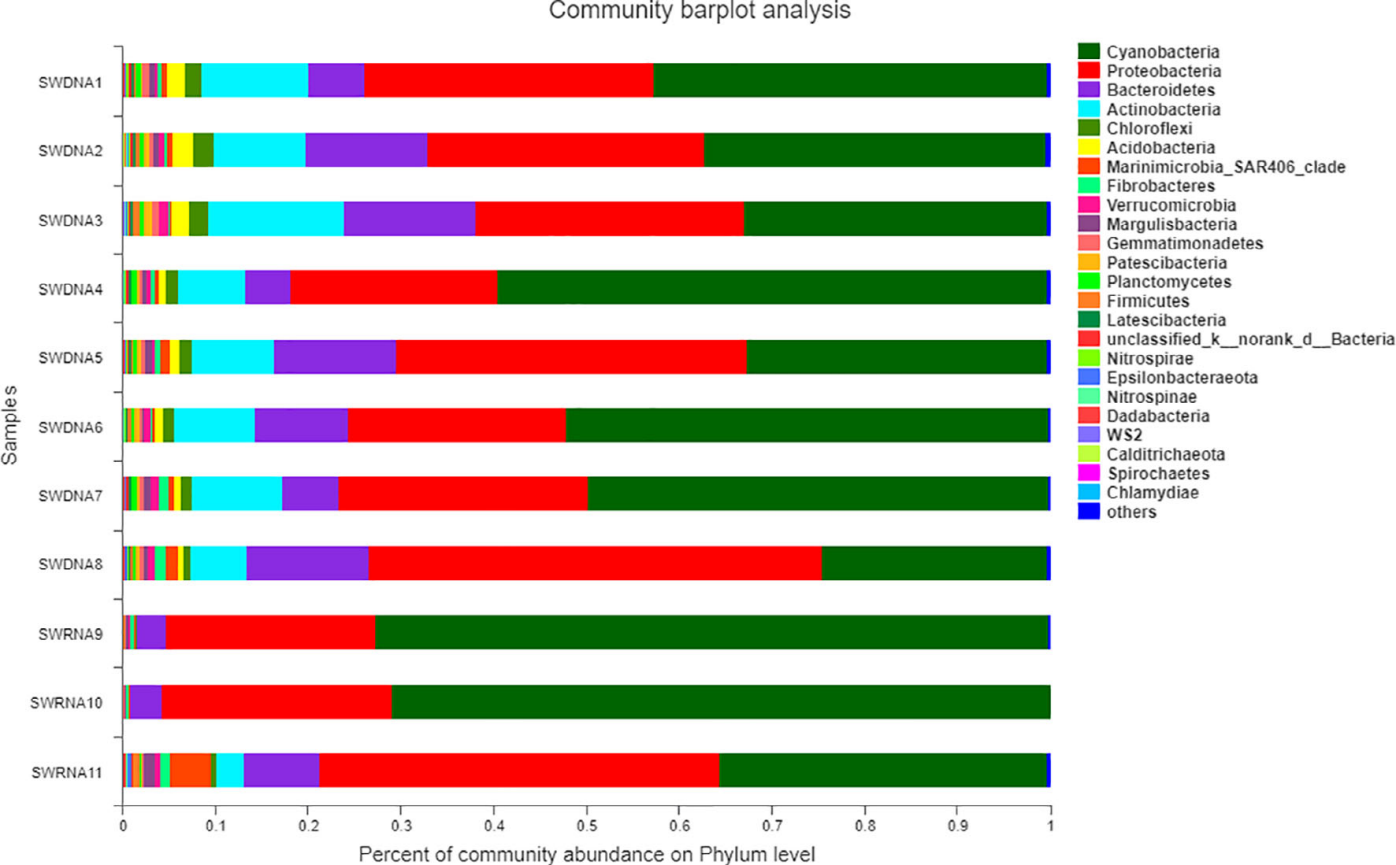
The gene sequence of bacteria in the SWDNA group and SWRNA group samples at the phylum level. Figure 1 showed that in the SWDNA group, *Cyanobacteria* were the most predominant phylum, accounting for 24.16%–58.87% of the total number of phyla, *Proteobacteria* represented the second most common phylum, accounting for 22.60%–49.18% of the total number of phyla, followed by *Bacteroides* at 4.81%–14.09%. In the SWRNA group, *Cyanobacteria*, *Proteobacteria*, and *Bacteroides* were also the most predominant phyla, with *Cyanobacteria* as the predominant phylum, accounting for 35.36%–72.62% of the total number of phyla, followed by *Proteobacteria* at 22.62%–43.09% and *Bacteroides* at 3.02%–8.22%.

In the SWDNA group, *Cyanobacteria* were the most predominant phylum, accounting for 24.16% to 58.87% of the total number of phyla. *Proteobacteria* represented the second most common phylum, accounting for 22.60% to 49.18% of the total number of phyla, followed by *Bacteroides* at 4.81% to 14.09%, Actinobacteria at 6.09% to 11.49%, *Chloroflexi* at 0.74% to 2.24%, and *Acidobacteria* at 0.71% to 2.27%. In the SWRNA group, *Cyanobacteria*, *Proteobacteria*, and *Bacteroides* were also the most predominant phyla, with *Cyanobacteria* as the predominant phylum, accounting for 35.36% to 72.62% of the total number of phyla, *Proteobacteria* at 22.62% to 43.09%, *Bacteroides* at 3.02% to 8.22%, followed by *Marinimicrobia_SAR406_clade* at 0.04% to 4.37%, *Actinobacteria* at 0.06% to 2.90%, and *Fibrobacteres* at 0.29% to 1.05% (Tables S2 and S3). Figure 1 reports the composition of the total bacterial community and active bacterial community and rank the phyla in the SWDNA and SWRNA groups by average percentage.

#### Class Level

**Fig 2:**
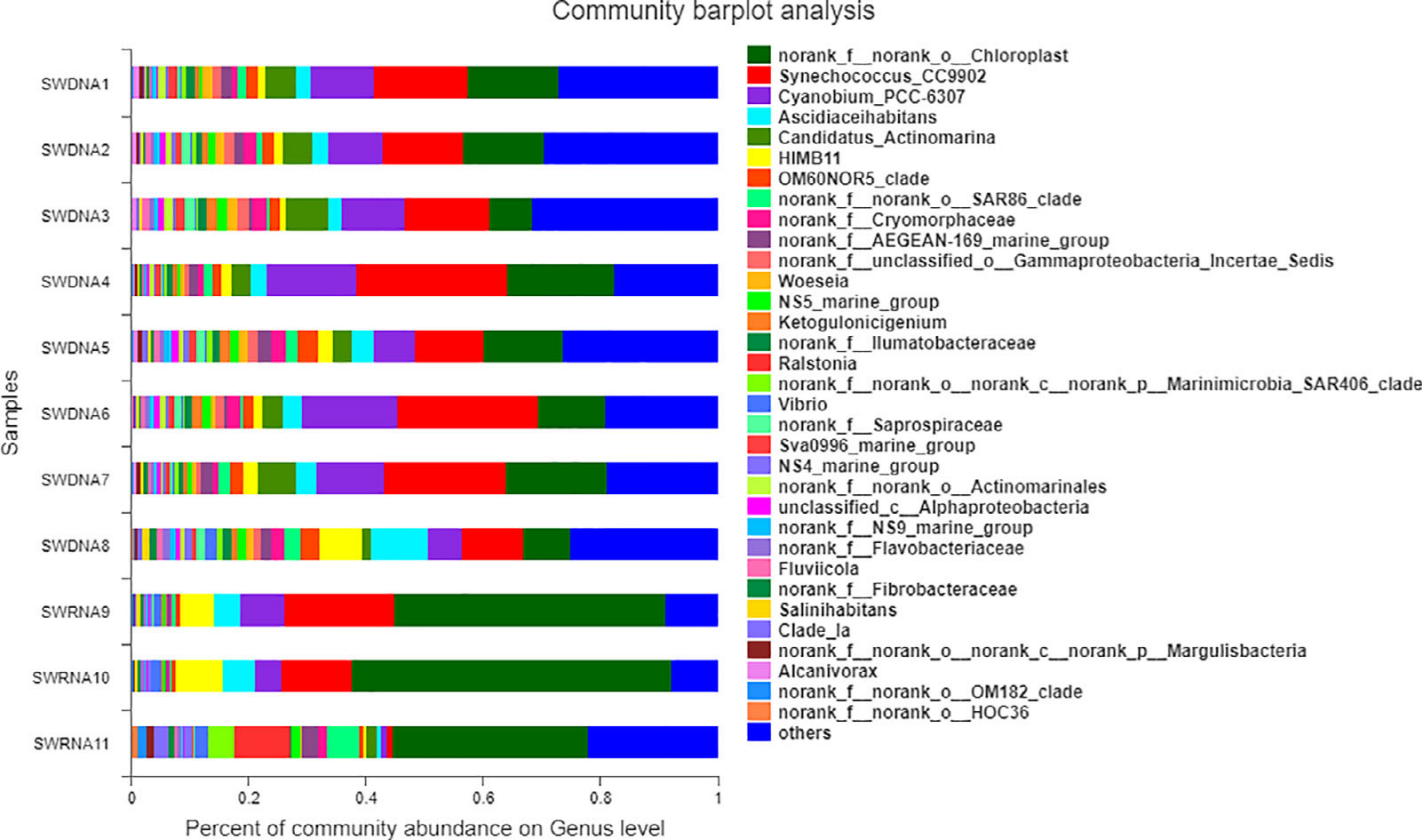
The gene sequence of bacteria in the SWDNA group and SWRNA group samples at the class level. Figure 2 showed that in the SWDNA group, at the class level, oxygen‐producing photobacteria comprised the predominant group, accounting for 24.15%–51.16%, followed by α‐proteobacteria at 11.04%–29.99% and γ‐proteobacteria at 8.97%–16.72%. In the SWRNA group, aerobic *Photobacteria* dominated, accounting for 35.31%–72.60% of the total number of phyla, followed by *α‐proteobacteria* at 12.55%–18.05% and *γ‐proteobacterium* at 6.02%–28.74%.

In the SWDNA group, at the class level, oxygen‐producing *photobacteria* comprised the most predominant group, accounting for 24.15% to 51.16%. *α‐proteobacteria* was the second most predominant class, accounting for 11.04% to 29.99%. *γ‐proteobacteria* was the third most predominant class, which accounted for 8.97% to 16.72%, followed by *Bacteroidia* at 4.63% to 13.56%, *Actinobacteria* at 6.09% to 13.56%, and *δ‐proteobacteria* at 2.10% to 4.31%. In the SWRNA group, *Oxyphotobacteria* dominated, accounting for 35.31% to 72.60% of the total number of phyla, followed by *α‐proteobacteria* at 12.55% to 18.05%, *γ‐proteobacterium* at 6.02% to 28.74%, *Bacteroidia* at 3.01% to 8.18%, *Marinimicrobia_SAR406_clade* at 0.04% to 4.37%, and *δ‐proteobacteria* at 0.67% to 1.80% (Tables S4 and S5). Figure 2 showed the rank of ocean bacterioplankton in class level, which revealed that *Oxyphotobacteria* was the most common class in the SWDNA and SWRNA groups at 40.82% and 59.59%, respectively.

#### Genus Level

**Fig 3:**
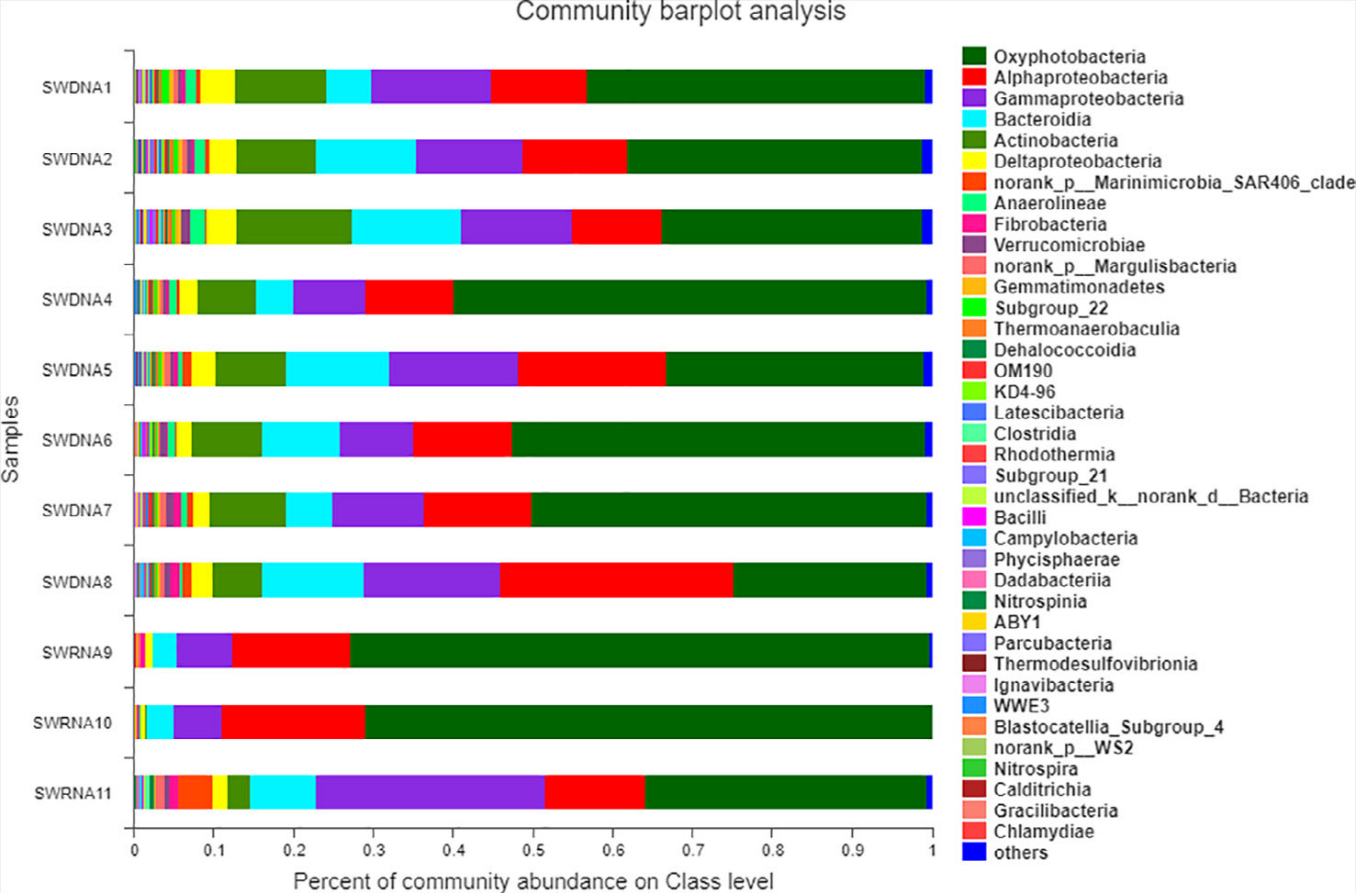
The gene sequence of bacteria in the SWDNA group and SWRNA group samples at the genus level. Figure 3 showed that in the SWDNA group, the six most common genera were *Synechococcus_CC9902* (17.03%), *Chloroplast* (13.07%), *Cyanobium_PCC‐6307* (10.70%), *Candidatus Actinomarina* (4.51%), *Ascidiaceihabitans* (3.77%), and *HIMB11* (2.37%), accounting for 51.45% of the total genera of which *Synechococcus_CC9902* was the main genus, accounting for 10.43%–25.94%, followed by *chloroplasts* at 7.20%–17.96% and *Candidatus_Actinomarina* at 5.73%–16.05%. In the SWRNA group, the six most common genera were *Chloroplast* (44.57%), *Synechococcus_CC9902* (10.61%), *HIMB11* (4.75%), *Cyanobium_PCC‐6307* (4.37%), *Ascidiaceihabitans* (3.57%), and *Ralstonia* (3.19%), accounting for 71.06% of the total genera.

In the SWDNA group, the six most common genera were *Synechococcus_CC9902* (17.03%), *Chloroplast* (13.07%), *Cyanobium_PCC‐6307* (10.70%), *Candidatus Actinomarina* (4.51%), *Ascidiaceihabitans* (3.77%), and *HIMB11* (2.37%), accounting for 51.45% of the total genera, of which *Synechococcus_CC9902* was the main genus, accounting for 10.43% to 25.94%, followed by *chloroplasts* at 7.20% to 17.96%, *Cyanobium_PCC‐6307* at 5.73% to 16.05%, *Candidatus Actinomarina* at 1.57% to 7.46%, *Ascidiaceihabitans* at 2.07% to 9.78%, and *HIMB11* at 1.00% to 7.34% (Table S6). In the SWRNA group, the six most common genera were *Chloroplast* (44.57%), *Synechococcus_CC9902* (10.61%), *HIMB11* (4.75%), *Cyanobium_PCC‐6307* (4.37%), *Ascidiaceihabitans* (3.57%), and *Ralstonia* (3.19%), accounting for 71.06% of the total genera of which *Chloroplast* was the most predominant genus accounting for 33.28% to 54.35%, *Synechococcus_CC9902* accounting for 1.06% to 18.80%, *HIMB11* at 0.55% to 7.95%, *Cyanobium_PCC‐6307* at 0.97% to 7.65%, *Ascidiaceihabitans* at 0.78% to 5.60%, and *Ralstonia* 0% to 9.58% (Table S7). Figure 3 revealed the rank of bacteria in genus level and *Synechococcus_CC9902* and *Chloroplast* were the most common genus in the SWDNA and SWRNA groups, respectively.

### Sample Comparative Analysis

**Fig 4:**
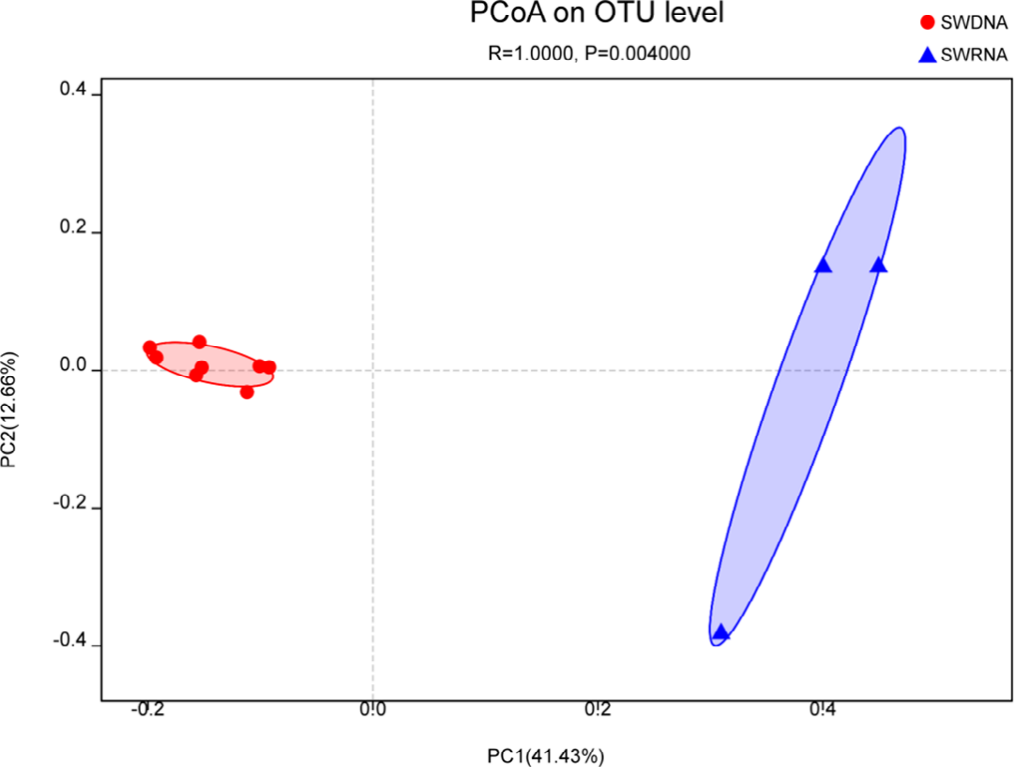
Principal coordinate analysis (PCoA) results of SWDNA group and SWRNA group. Figure 4 showed that using PCoA in beta diversity analysis to statistically analyze the differences in the structure and composition of SWDNA and SWRNA groups, based on the analysis of differences between groups displayed by the unweighted‐unifrac distance matrix (ANOSIM), SWDNA and SWRNA groups were divided into two clusters (R = 1; *P* = 0.004).

Using PCoA in beta diversity analysis to statistically analyze the differences in the structure and composition of SWDNA and SWRNA groups (Fig. 4), based on the analysis of differences between groups displayed by the unweighted‐unifrac distance matrix (ANOSIM), SWDNA and SWRNA groups were divided into two clusters (R = 1; *P* = 0.004). It shows that there were obvious differences between the total bacterial community and the active bacterial community in ocean bacterioplanktons. The algorithm did not consider the level of species abundance, but only considered the evolutionary relationship of bacteria, and the dominant bacterioplanktons between two groups were relatively consistent. Hence, the difference mainly derives from low‐abundance bacterial species, rather than from predominant bacterial species.

**Fig 5:**
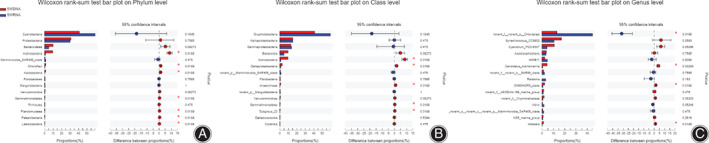
The difference test of the active bacterial communities of SWDNA and SWRNA groups at the phylum, class, and genus level respectively. (A) **(phylum level)** showed that at the phylum level, *Actinomycetes* was more abundant in SWDNA group (*P* < 0.05). (B) **(class level)** showed that at the class level, *Actinomycetes* (*δ‐ Proteus*) was more abundant in SWDNA group (*P* < 0.05). (C) **(genus level)** showed that at the genus level, *Chloroplast* was more abundant in SWRNA group (*P* < 0.05).

The species abundance differences were estimated by Wilcoxon rank‐sum test to evaluate the differences in specific species among SWDNA and SWRNA groups. It revealed that at the phylum level, *Actinomycetes* was more abundant in SWDNA group (9.17 *vs* 1.02%, *P* < 0.05) (Fig. 5A); at the class level, *Actinomycetes* (δ‐ Proteus) was more abundant in SWDNA group (9.47 *vs* 1.01%, *P* < 0.05) (Fig. 5B); and at the genus level, *Chloroplast* was more abundant in SWRNA group (13.07 *vs* 44.57%, *P* < 0.05) (Fig. 5C).

### Culture and Identification of the Bacterioplankton

A total of 53 strains of bacteria were isolated in eight culture media, the separate results were shown in Fig. S1. In summary, *Vibrio alginolyticus* had the highest proportion, accounting for 15.10%. The second predominant bacteria was *Enterobacter cloacae* (13.22%). The detection rates of *Vibrio parahaemolyticus*, *Vibrio fluvialis*, and *Pseudomonas aeruginosa* were both 11.32%, followed by *Klebsiella pneumoniae*, *Proteus vulgaris*, *Photobacterium mermaid*, and *Shewanella seaweed*, in which all of them accounted for 9.43% (Table S8). Figure S1 showed the bacterial culture results, from A to H are blood agar, MacConkey agar, chocolate agar, Chinese blue agar, SS agar, TCBS agar, Baird‐Parker agar, and 3% sodium chloride tryptone soy agar agar, which represented *Vibrio*, *Enterobacter*, non‐fermentative bacteria, *Staphylococci*, Gram‐positive *bacilli*, *Enterobacter*, *Vibrio*, *Staphylococcus*, and *Vibrio parahaemolyticus*.

## Discussion

This is the first study to explore the physicochemical characteristics of seawater and bacterial community composition in the coastal zone of Changle city, using a combination of new generation high‐throughput sequencing technology and bacterial culture. Illumi MiSeq sequencing of seawater bacteria revealed that the total bacterial community groups and the active bacterial community groups mainly comprised *Cyanobacteria*, *Proteobacteria*, and *Bacteroides* at the phylum level; *Oxyphotobacteria*, *α‐Proteobacteria*, and *γ‐Proteobacteria* at the class level; with *Synechococcus_CC9902*, *Chloroplast*, and *Cyanobium_PCC‐6307* comprising the predominant genera. In addition, the main significant difference between the total bacterial community and the active bacterial community in seawater was manifested in low‐abundance bacteria.

### Effects of Different Physicochemical Characteristics of Seawater on Human Body

The physicochemical characteristics of seawater differ based on location. For instance, the sodium ion content of the sea water in the Gulf of Aden is twice that of the human body, and the osmotic pressure is 3.5 times that of the human body^40^. In contrast, both the sodium ion content and osmotic pressure of the seawater from the present study are lower than those of the seawater in the Gulf of Aden. Many factors affect the physicochemical characteristics of seawater, including temperature, monsoon season, latitude, and the specific geography^41^, ^42^. In the Gulf of Aden, which is a tropical sea area, seawater temperature is 25–31°C, while the annual average seawater temperature in the sea area of the present study was 18–21°C. Accordingly, the difference in seawater temperature and geographical position between the coastal zone of Changle city and the Gulf of Aden may lead to different physical and chemical properties in seawater.

Hypertonic seawater affects the metabolism of various cytokines and damages the body, including damage to human cell membranes. The alkaline environment also strengthens the damage response of cells, dissolves the cells, releases membrane phospholipids, activates the oxidative stress response, and leads to cytolysis^43^, ^44^. Therefore, to formulate more targeted treatment plans, wounds immersed in seawater should be considered for the damaging effects of seawater's high sodium, hypertonicity, and partial alkalinity on human cells and tissues. A previous study has reported that compared with immersing in freshwater, immersing in seawater will increased the serious degree of lung injury^45^.

### The Predominant Community Composition of Bacterioplankton by Illumina MiSeq Sequencing and its Characteristics

In this study, the predominant species of planktonic bacteria in seawater were similar to the composition of global bacterial species^46^, ^47^, ^48^, mainly comprising *oxygen‐producing photobacteria*, *α‐proteobacteria*, and *γ‐proteobacteria*. These three predominant bacterial groups in the sea area of this study are all of the algae bacteria genus, as well as *Cyanophyta oxyphotobacteria*. These findings are consistent with those of the Florida sea area, where the most abundant bacteria in southeast Florida was *Cyanobacteria* ^11^. However, it contrasts with other studies: *Cyanobacteria* accounted for 50% of the total flora in the current study, whereas previous studies reported that the *Proteus* phylum accounted for the largest proportion, with *Cyanophyta* only accounting for about 1%–10%^7^, ^8^. *Cyanobacteria* plays an active role in sewage treatment and water self‐purification, and they can be used to improve the soil environment and produce extracellular pesticides^49^. Additionally, there is no evidence that *Cyanobacteria* are causative pathogens of limb infections after injuries in seawater. However, there were obvious differences between the complex total bacterial communities and active bacterial communities in the ocean. The difference mainly derives from low‐abundance bacterial species, rather than from predominant bacterial species^50^, ^51^.

### The Predominant Species of Bacterial Culture and its Characteristic

In the present study, at the species level, *Vibrio alginolyticus*, *Vibrio parahaemolyticus*, and *Vibrio fluvialis* were the predominant species based on bacterial culture. As an opportunistic pathogen to both humans and marine animals, *Vibrio alginolyticus* can cause lung and liver damage in healthy mice^52^. *Vibrio parahaemolyticus* can accumulate at high levels in shellfish, and human consumption of bacteria‐infected oysters can cause gastrointestinal infections^53^. The hemolysin of *Vibrio fluvialis* induces the secretion of interleukin‐1β through the activation of the NLRP3 inflammasome, which activates the immune response of the infected individual^54^.

*Vibrio* was the most predominant genus upon bacterial culture, while the proportion of *Vibrio* was only the eighth most common genus based on the results of Illumina MiSeq sequencing. This difference in results may be because bacterial culture is easily restricted by external conditions and environment and cannot distinguish the advantages and proportions of complex bacterial samples; in contrast, Illumina MiSeq sequencing can more accurately and comprehensively detect specific live bacterial species and proportions through reverse transcription of live bacterial cDNA.

### Limitations of this Study

There were some limitations in this study. First, the seawater sampling location was only from one city, and follow‐up research will expand the seawater sampling range and it will greatly improve our knowledge of the ocean bacterial community structure. Second, Illumina MiSeq sequencing only detects genus‐level data. Therefore, it may show irregularities in the bacterial composition at the species level. However, based on the bacterial composition at the genus level, the predominant species at the species level can also be indirectly determined.

### Conclusion

This study applied Illumina MiSeq sequencing in combination with bacterial culture techniques to provide an in‐depth study of the types and composition of bacterioplankton in the coastal zone of Changle city. The high‐abundance bacteria at the phylum level of the total seawater bacterial community group and seawater active bacterial community group consisted of *Cyanobacteria*, *Proteobacteria*, and *Bacteroidetes*; and the most common genera was *Synechococcus_CC9902*, *Chloroplast*, and *Cyanobium_PCC‐6307*. Exploring the composition and differences of seawater bacteria can not only effectively reduce the prevalence of infectious diseases caused by seawater bacteria, but also have important significance for the diagnosis and treatment of seawater‐related bacterial infections.

## Authorship Declaration

All authors listed meet the authorship criteria according to the latest guidelines of the International Committee of Medical Journal Editors, and that all authors are in agreement with the manuscript.

## Supporting information


**Figure S1** Test chart of seawater filter membrane after bacteria culture. Figure S1 showed that A‐H are as follows: blood agar, MacConkey agar, chocolate agar, Chinese blue agar, SS agar, TCBS agar, Baird‐Parker agar, and 3% sodium chloride tryptone soy agar agar.

**Table S1** Environmental factor indicators for collecting seawater.
**Table S2** The composition of the microbial community at the phylum level of the total bacterial group in seawater.
**Table S3** The composition of the microbial community at the phylum level of active bacteria in seawater.
**Table S4** The composition of the microbial community of the total bacterial group in seawater at the class level.
**Table S5** Composition of the microbial community at the class level of active bacteria in seawater.
**Table S6** The composition of the microbial community at the genus level of the total bacterial group in seawater.
**Table S7** Composition of microbial community at the genus level of active bacteria in seawater.
**Table S8** Summary results of bacterial plate culture detection in eight seawater samples.
