# Supplementary figures and images for: Analysis of Community Composition of Bacterioplankton in Changle Seawater in China by Illumina Sequencing Combined with Bacteria Culture
Source: Orthop Surg. 2021 Nov 24;14(1):139–48. doi: 10.1111/os.13060 (PMC8755877; doi:10.1111/os.13060)

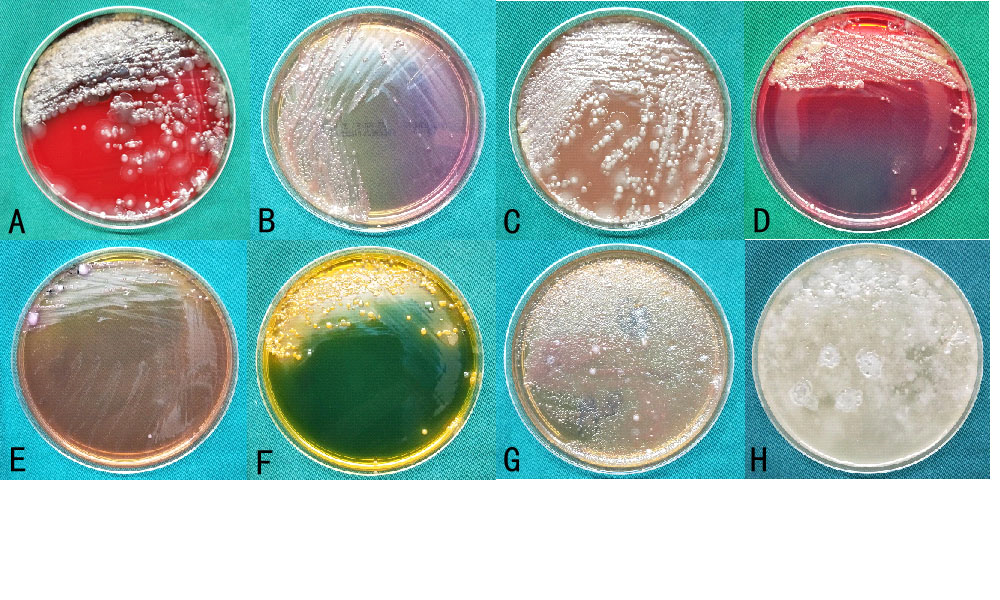

Supplement: Supplementary file 1 — Figure S1 Test chart of seawater filter membrane after bacteria culture. Figure S1 showed that A‐H are as follows: blood agar, MacConkey agar, chocolate agar, Chinese blue agar, SS agar, TCBS agar, Baird‐Parker agar, and 3% sodium chloride tryptone soy agar agar. [file OS-14-139-s002.tif]
