# Supplementary material for: Analysis of Community Composition of Bacterioplankton in Changle Seawater in China by Illumina Sequencing Combined with Bacteria Culture
Source: Orthop Surg. 2021 Nov 24;14(1):139–48. doi: 10.1111/os.13060 (PMC8755877; doi:10.1111/os.13060)
Supplement: Supplementary file 2 — Table S1 Environmental factor indicators for collecting seawater. Table S2 The composition of the microbial community at the phylum level of the total bacterial group in seawater. Table S3 The composition of the microbial community at the phylum level of active bacteria in seawater. Table S4 The composition of the microbial community of the total bacterial group in seawater at the class level. Table S5 Composition of the microbial community at the class level of active bacteria in seawater. Table S6 The composition of the microbial community at the genus level of the total bacterial group in seawater. Table S7 Composition of microbial community at the genus level of active bacteria in seawater. Table S8 Summary results of bacterial plate culture detection in eight seawater samples. [file OS-14-139-s001.docx]

Supplement Table1. Environmental factor indicators for collecting seawater

| Environmental factor index | Along the coast | Laboratory |
| --- | --- | --- |
| Temperature (℃) | 28.6±1.3 | 25.0±0.5 |
| Water temperature (℃) | 29.1±3.2 | 28.5±1.4 |
| PH | 8.0316±0.4 | 8.0316±0.4 |
| Na^+^（mmol/L） | 325 | 325 |
| Cl^-^（mmol/L） | 378 | 378 |
| K^+^（mmol/L） | 8.3 | 8.3 |
| Ca^2+^（mmol/L） | 9.1 | 9.1 |
| Osmotic pressure (mmol/L) | 725 | 725 |
| transparency | Turbid | Clearer |
| Sunshine (h) | 6 | — |
| Wind power (level) | 2-3 | — |
| Wave intensity | Light waves | — |
| Distance between seawater collection and shore (m) | 10 | — |
| Time from seawater collection to processing (h) | 2 | — |

Supplement Table 2. The composition of the microbial community at the phylum level of the total bacterial group in seawater

| Phylogeny level | SWDNA1 | SWDNA2 | SWDNA3 | SWDNA4 | SWDNA5 | SWDNA6 | SWDNA7 | SWDNA8 | Average percentage |
| --- | --- | --- | --- | --- | --- | --- | --- | --- | --- |
| Cyanobacteria | 41.86 | 36.53 | 32.55 | 58.87 | 31.84 | 51.19 | 49.74 | 24.16 | 40.86 |
| Proteobacteria | 31.12 | 29.92 | 28.87 | 22.60 | 38.50 | 23.83 | 26.86 | 49.18 | 31.34 |
| Bacteroidetes | 6.16 | 13.11 | 14.09 | 4.81 | 13.01 | 10.29 | 6.02 | 12.91 | 10.01 |
| Actinobacteria | 11.49 | 9.66 | 14.62 | 7.24 | 8.58 | 8.59 | 9.49 | 6.09 | 9.17 |
| Chloroflexi | 2.01 | 2.24 | 2.11 | 1.40 | 1.47 | 1.26 | 1.35 | 0.74 | 1.56 |
| Acidobacteria | 1.95 | 2.27 | 1.94 | 0.78 | 1.09 | 1.06 | 0.71 | 0.70 | 1.31 |

Supplement Table 3. Composition of the microbial community at the phylum level of active bacteria in seawater

| Phylogeny level | SWRNA9 | SWRNA10 | SWRNA11 | Average percentage |
| --- | --- | --- | --- | --- |
| Cyanobacteria | 72.62 | 70.86 | 35.36 | 59.61 |
| Proteobacteria | 22.62 | 24.74 | 43.09 | 30.15 |
| Bacteroidetes | 3.02 | 3.40 | 8.22 | 4.88 |
| Marinimicrobia_SAR406_clade | 0.08 | 0.04 | 4.37 | 1.50 |
| Actinobacteria | 0.11 | 0.06 | 2.90 | 1.02 |
| Fibrobacteres | 0.44 | 0.29 | 1.05 | 0.59 |

Supplement Table 4. The composition of the microbial community of the total bacterial group in seawater at the class level

| SWDNA group level bacterial species | SWDNA1 | SWDNA2 | SWDNA3 | SWDNA4 | SWDNA5 | SWDNA6 | SWDNA7 | SWDNA8 | Average percentage |
| --- | --- | --- | --- | --- | --- | --- | --- | --- | --- |
| Oxyphotobacteria | 41.85 | 36.52 | 32.53 | 58.84 | 31.81 | 51.16 | 49.73 | 24.15 | 40.82 |
| Alphaproteobacteria | 11.77 | 13.25 | 11.43 | 11.04 | 18.94 | 12.76 | 13.41 | 29.99 | 15.32 |
| Gammaproteobacteria | 15.03 | 13.16 | 13.65 | 9.27 | 16.17 | 8.97 | 11.32 | 16.72 | 13.04 |
| Bacteroidia | 5.81 | 12.63 | 13.56 | 4.63 | 12.67 | 10.05 | 5.83 | 12.68 | 9.73 |
| Actinobacteria | 11.49 | 9.66 | 14.62 | 7.24 | 8.58 | 8.59 | 9.49 | 6.09 | 9.47 |
| Deltaproteobacteria | 4.31 | 3.51 | 3.79 | 2.28 | 3.38 | 2.10 | 2.11 | 2.45 | 2.99 |

Supplement Table 5. Composition of the microbial community at the class level of active bacteria in seawater

| SWRNA group level bacterial species | SWRNA9 | SWRNA10 | SWRNA11 | Average percentage |
| --- | --- | --- | --- | --- |
| Oxyphotobacteria | 72.60 | 70.86 | 35.31 | 59.59 |
| Alphaproteobacteria | 14.93 | 18.05 | 12.55 | 15.18 |
| Gammaproteobacteria | 6.89 | 6.02 | 28.74 | 13.88 |
| Bacteroidia | 3.01 | 3.40 | 8.18 | 4.86 |
| Marinimicrobia_SAR406_clade | 0.08 | 0.04 | 4.37 | 1.50 |
| Deltaproteobacteria | 0.80 | 0.67 | 1.80 | 1.09 |

Supplement Table 6. The composition of the microbial community at the genus level of the total bacterial group in seawater

| SWDNA group genus level bacterial classification | SWDNA1 | SWDNA2 | SWDNA3 | SWDNA4 | SWDNA5 | SWDNA6 | SWDNA7 | SWDNA8 | Average percentage |
| --- | --- | --- | --- | --- | --- | --- | --- | --- | --- |
| Synechococcus_CC9902 | 15.74 | 13.72 | 14.34 | 25.94 | 11.52 | 23.73 | 20.84 | 10.43 | 17.03 |
| Chloroplast | 15.72 | 13.52 | 7.20 | 17.96 | 13.42 | 11.34 | 17.40 | 7.98 | 13.07 |
| Cyanobium_PCC-6307 | 10.37 | 9.25 | 10.97 | 14.92 | 6.87 | 16.05 | 11.47 | 5.73 | 10.70 |
| Candidatus_Actinomarina | 5.48 | 5.00 | 7.46 | 3.52 | 3.13 | 3.60 | 6.29 | 1.57 | 4.51 |
| Ascidiaceihabitans | 2.15 | 2.74 | 2.07 | 2.71 | 3.95 | 3.18 | 3.55 | 9.78 | 3.77 |
| HIMB11 | 1.14 | 1.45 | 1.00 | 1.47 | 2.70 | 1.42 | 2.45 | 7.34 | 2.37 |

Supplement Table 7. Composition of microbial community at the genus level of active bacteria in seawater

| SWRNA group genus level bacterial classification | SWRNA9 | SWRNA10 | SWRNA11 | Average percentage |
| --- | --- | --- | --- | --- |
| norank_f__norank_o__Chloroplast | 46.09% | 54.35% | 33.28% | 44.57% |
| Synechococcus_CC9902 | 18.80% | 11.98% | 1.06% | 10.61% |
| HIMB11 | 5.76% | 7.95% | 0.55% | 4.75% |
| Cyanobium_PCC-6307 | 7.65% | 4.48% | 0.97% | 4.37% |
| Ascidiaceihabitans | 4.34% | 5.60% | 0.78% | 3.57% |
| Ralstonia | 0 | 0 | 9.58% | 3.19% |

Supplement Table 8. Summary results of bacterial plate culture detection in 8 seawater samples

| Bacteria (genus) | Strain | Number of detected plants (n) | Detection rate(%) |
| --- | --- | --- | --- |
| Vibrio | Vibrio alginolyticus | 8 | 15.10 |
|  | Vibrio parahaemolyticus | 6 | 11.32 |
|  | Vibrio fluvialis | 6 | 11.32 |
| Enterobacteriaceae | Enterobacter cloacae | 7 | 13.22 |
|  | Klebsiella pneumoniae | 5 | 9.43 |
|  | Proteus vulgaris | 5 | 9.43 |
|  | Photobacterium mermaid | 5 | 9.43 |
| Non-fermenting bacteria | Pseudomonas aeruginosa | 6 | 11.32 |
|  | Shewanella seaweed | 5 | 9.43 |
| Total |  | 53 | 100.00 |
